# miR-129-3p controls centrosome number in metastatic prostate cancer cells by repressing CP110

**DOI:** 10.18632/oncotarget.7572

**Published:** 2016-02-23

**Authors:** Irene V. Bijnsdorp, Jasmina Hodzic, Tonny Lagerweij, Bart Westerman, Oscar Krijgsman, Jurjen Broeke, Frederik Verweij, R. Jonas A. Nilsson, Lawrence Rozendaal, Victor W. van Beusechem, Jeroen A. van Moorselaar, Thomas Wurdinger, Albert A. Geldof

**Affiliations:** ^1^ Department of Urology, VU University Medical Center, Amsterdam, The Netherlands; ^2^ Department of Medical Oncology, VU University Medical Center, Amsterdam, The Netherlands; ^3^ Department of Neurosurgery, VU University Medical Center, Amsterdam, The Netherlands; ^4^ Department of Pathology, VU University Medical Center, Amsterdam, The Netherlands; ^5^ Department of Molecular Oncology, The Netherlands Cancer Institute, Amsterdam, The Netherlands; ^6^ Center for Neurogenomics and Cognitive Research, VU University, Amsterdam, The Netherlands; ^7^ Department of Radiation Sciences, Oncology, Umeå University, Umeå, Sweden; ^8^ Department of Neurology, Massachusetts General Hospital, Harvard Medical School, Boston, MA, USA

**Keywords:** prostate cancer, centrosome, CP110, miR-129-3p, metastasis

## Abstract

The centrosome plays a key role in cancer invasion and metastasis. However, it is unclear how abnormal centrosome numbers are regulated when prostate cancer (PCa) cells become metastatic. CP110 was previously described for its contribution of centrosome amplification (CA) and early development of aggressive cell behaviour. However its regulation in metastatic cells remains unclear. Here we identified miR-129-3p as a novel metastatic microRNA. CP110 was identified as its target protein. In PCa cells that have metastatic capacity, CP110 expression was repressed by miR-129-3p. High miR-129-3p expression levels increased cell invasion, while increasing CP110 levels decreased cell invasion. Overexpression of CP110 in metastatic PCa cells resulted in a decrease in the number of metastasis. In tissues of PCa patients, low CP110 and high miR-129-3p expression levels correlated with metastasis, but not with the expression of genes related to EMT. Furthermore, overexpression of CP110 in metastatic PCa cells resulted in excessive-CA (E-CA), and a change in F-actin distribution which is in agreement with their reduced metastatic capacity. Our data demonstrate that miR-129-3p functions as a CA gatekeeper in metastatic PCa cells by maintaining pro-metastatic centrosome amplification (CA) and preventing anti-metastatic E-CA.

## INTRODUCTION

Prostate cancer (PCa) is the second most common cause of cancer related death in males in the Western countries [1]. For patients with metastatic PCa no curative therapy is available. The progression of localized PCa to metastatic variants involves multiple sequential steps, including cell migration and invasive growth [2].

The centrosome is an organelle that serves as the main microtubule organizing centre of the cell [3]. Prostate cancer cells frequently show centrosome amplification (CA; 3-5 centrosomes/cell). CA leads to promotion of tumor growth through induction of chromosomal missegregation and modulation of the microtubule cytoskeleton, causing enhanced directional migration and invasion of malignant cells [4–6]. CA conveys cytoskeletal advantages that enhance cell polarization, Golgi-dependent vesicular trafficking, and stromal invasion [4–6]. CP110 is an evolutionary conserved centrosomal protein important for centrosome functioning [7–9], controlling cell migration [10]. CP110 overexpression caused CA and increased the invasive phenotype of cells [7, 11]. However, the actual contribution of CP110 in PCa metastasis has not been described.

MicroRNAs (miRNAs) are small non-coding RNAs that suppress translation of target mRNAs, and tightly regulate numerous cellular processes [12]. In cancer cells miRNAs are often deregulated [13], including in PCa cells [14, 15]. Multiple studies identified miRNAs correlating to PCa metastasis or PCa survival [16–18]. In non-neoplastic cells, CP110 expression can be regulated by miR-34/449 [19] and miR-129-3p [10]. To determine the potential contribution of miRNAs in PCa cells to the regulation of CP110 and metastasis, we performed miRNA profiling of metastatic and non-metastatic PCa tissues. We confirmed the correlative expression of miRNAs previously associated with metastatic PCiling of metastatic and non-metastatic PCa tissues. We confirmed the correlative expression of miRNAs previously associated with metastatic PCa, including miR-34 [17]. Moreover we observed high expressiojn of miR-129-3p in metastatic PCa cells. MiR-129-3p has not been previously associated with PCa metastasis. MiR-129-3p decreased the expression of the centrosomal protein CP110. Lack of CP110 inhibition led to excessive centrosome amplification (E-CA; > 5 centrosomes/cell), increased E-cadherin expression, deregulation of the F-actin cytoskeleton and diminished invasion and metastasis. We show that centrosome regulation in metastatic PCa cells differs from that in early stages of prostate cancer development. When cells acquire a metastatic capacity, CP110 levels are reduced to control centrosome number and thereby prevent E-CA.

## RESULTS

### miR-129-3p is overexpressed in metastatic PCa

**Figure 1:**
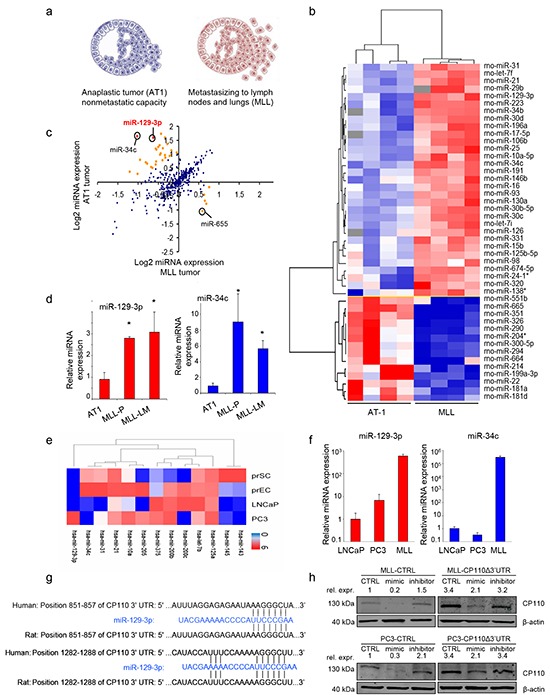
miR-129-3p is overexpressed in metastatic PCa **a.** Characteristics of the selected variants of the PCa Dunning progression model. **b.** Hierarchical cluster analysis of miRNA expression (miRNA microarray) of AT1 and MLL tumor tissues. **c.** Comparative expression analysis (microarray) between AT1 and AT1 and MLL tumor tissues. Each dot indicates one miRNA showing the average log2 expression value from four independent tissue samples. Red indicates >4 fold change, orange indicates 2-4 fold change, p<0.05. **d.** RT-PCR validation of miRNA expression levels of miR-129-3p and miR-34c in the tumor tissues of AT1 and MLL. Values represent means of three independent tumor tissue samples ± s.e.m. MLL-P = MLL primary tissue; MLL-LM = MLL lung metastasis tissue. **e.** RNAseq analysis of selected miRNAs in *in vitro* cultures of non-cancerous prSC, prEC and the PCa cell lines PC3 and LNCaP. Clustering of normalized, log transformed RNAseq expression data was performed using Cluster 3.0 software and visualized using Java treeview **f.** Relative miRNA expression levels of miR-129-3p and miR-34c in LNCaP, PC3 and MLL cells. Values represent means of three independent experiments ± s.e.m. **g.** Confirmed CP110 3′-UTR binding sites for miR-129-3p. **h.** CP110 protein expression in cells transfected with a CP110Δ'3UTR rescue construct, with or without transfection with miR-129-3p mimics or inhibitors. *p<0.05, T-test.

To identify miRNAs involved in PCa metastasis, we used the Dunning rat PCa progression model [20]. Two successive stages of progressive PCa were selected, i.e. AT-1 (AT1, locally invasive PCa), and MatLyLu (MLL, metastatic PCa) (Figure 1a). RNA was isolated from AT1 and MLL PCa tissues and miRNA expression array analyses were performed to determine which miRNAs are differentially expressed (Supplementary Table S1). The obtained miRNA expression profiles were subjected to (unsupervised) hierarchical cluster analyses (Figure 1b-1c). Only one miRNA (miR-665; Supplementary Table S1) was downregulated in metastatic MLL as compared to locally invasive AT1 PCa tissues (>2-fold, p<0.05). In contrast, in MLL tissues we identified in total 24 upregulated miRNAs (>2-fold, p<0.05; Supplementary Table S1). The pro-metastatic miRNAs that were increased most include the previously identified miR-34c [17], and a newly identified miRNA, miR-129-3p (Figure 1c; Supplementary Table S1). RT-PCR was used to validate the differential expression levels of pro-metastatic miR-129-3p and miR-34c (3- and 9-fold upregulation in MLL compared to AT-1 tumor tissues, respectively), (Figure 1d). Of note, the miR-129-3p and miR-34c levels in rat MLL lung metastases (MLL-LM) were similar to the levels in rat MLL primary PCa tissue (MLL-P). In addition, we performed RNAseq on non-neoplastic human prostate epithelial cells (prEC) and prostate fibroblasts (prSC) and the human PCa cell cultures LNCaP and PC3 (Figure 1e). LNCaP and PC3 are both cell lines derived from metastasis, however when grown as subcutaneous xenografts they do not demonstrate metastatic capacity [21]. Although PC3 in other systems, such as after intracardiac injection or injection into bone can be used to study metastasis [22], the miRNA expression of PC3 is consistent with the non-metastatic potential of PC3 when grown in subcutaneous models, i.e. low miR-34c and miR-129-3p expression. Expression of these miRNAs was confirmed by RT-PCR in cultured human LNCaP, PC3 and rat MLL PCa cells (Figure 1f). Previously, miR-129-3p was shown to functionally repress the translation of the centrosomal protein CP110. Because this miRNA has not been described before in metastatic PCa, it was highly differential, and is potentially related to CP110, we selected this miRNA for further evaluation. Figure 1g shows the two complementary binding sites of miR-129-3p in the 3′-UTR of the human and rat CP110 mRNAs. To validate that miR-129-3p represses CP110 in PCa cells, we overexpressed miR-129-3p in human PC3 and rat MLL cells by transfection of a miR-129-3p mimic. We indeed measured a reduction in CP110 protein levels by Western blot. Conversely, inhibition of miR-129-3p by transfection of anti-sense molecules resulted in increased CP110 protein levels (Figure 1h, left panel). In addition, we transfected PC3 and MLL cells with a CP110Δ3′UTR expression cassette without miR-129-3p 3′-UTR. Because the miR-129-3p binding site is located on the 3′-UTR this construct enables CP110 expression independently of miR-129-3p (Figure 1h, right panel). In these cells, CP110 expression maintained after transfection with either miR-129-3p mimic or inhibitor, with a modest modulation which is likely caused by endogenous CP110 expression. We performed a target scan using multiple miRNA-target databases, showing a strong putative match between highly expressed miRNAs and predicted target sequences in the 3′UTR of CP110. A total of 12/24 miRNAs (50%) that were upregulated in metastatic MLL tumors were predicted to have a potential binding site in CP110 (Supplementary Table S2).

### CP110 is repressed by miR-129-3p and downregulated in metastatic PCa

**Figure 2:**
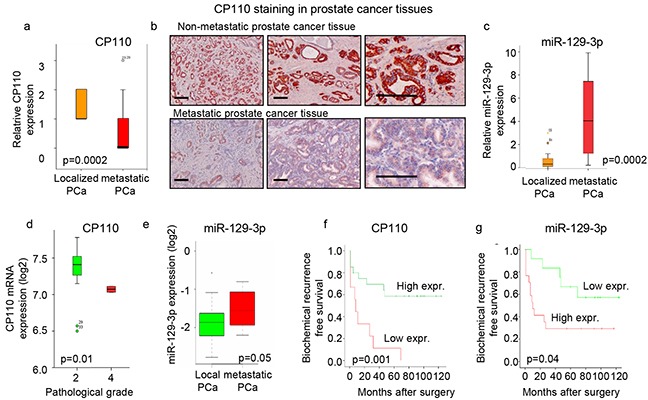
CP110 is repressed by miR-129-3p and downregulated in metastatic PCa **a.** Quantification of CP110 protein expression in tissues from 24 patients with localized PCa and 17 metastatic PCa. Y-axis represent relative expression based on intensity score of 0-3. **b.** Immunostaining of CP110 protein in prostate tissues. Scale bar, 200 μm. **c.** miR-129-3p expression in tissues from patients with localized PCa and metastatic PCa. **d.** CP110 mRNA expression from EXPO dataset. **e.** miR-129-3p expression in prostate tissues from the Martens-Uzunova dataset [17]. **f.** Expression of CP110 (score <1) correlated to time to biochemical recurrence. **g.** miR-129-3p expression (expression >4) correlated to time to biochemical recurrence.

The clinical relevance of miR-129-3p, and its target protein CP110 in PCa was determined by measuring their expression levels in human PCa tissues. CP110 protein expression levels were determined in tissues of locally confined PCa (n=24) and PCa tissues from patients with metastatic disease (n=17) obtained by radical prostatectomy (Figure 2a-2b), showing significant downregulation of CP110 in metastatic PCa tissues (p<0.0002) compared to local PCa tissues. Furthermore, miR-129-3p expression levels were significantly increased in metastatic PCa as compared to localized PCa tissues (p<0.0002) (Figure 2c). The increase in miR-129-3p (p<0.05) and decrease in CP110 (p<0.01) expression levels in metastatic PCa were validated in independent PCa datasets [17, 23–26] (Figure 2d-2e). Of note, the miR-129-3p and CP110 expression levels inversely correlated with the time to biochemical PCa recurrence after surgery (Figure 2f-2g).

### miR-129-3p and CP110 regulate PCa metastasis

**Figure 3:**
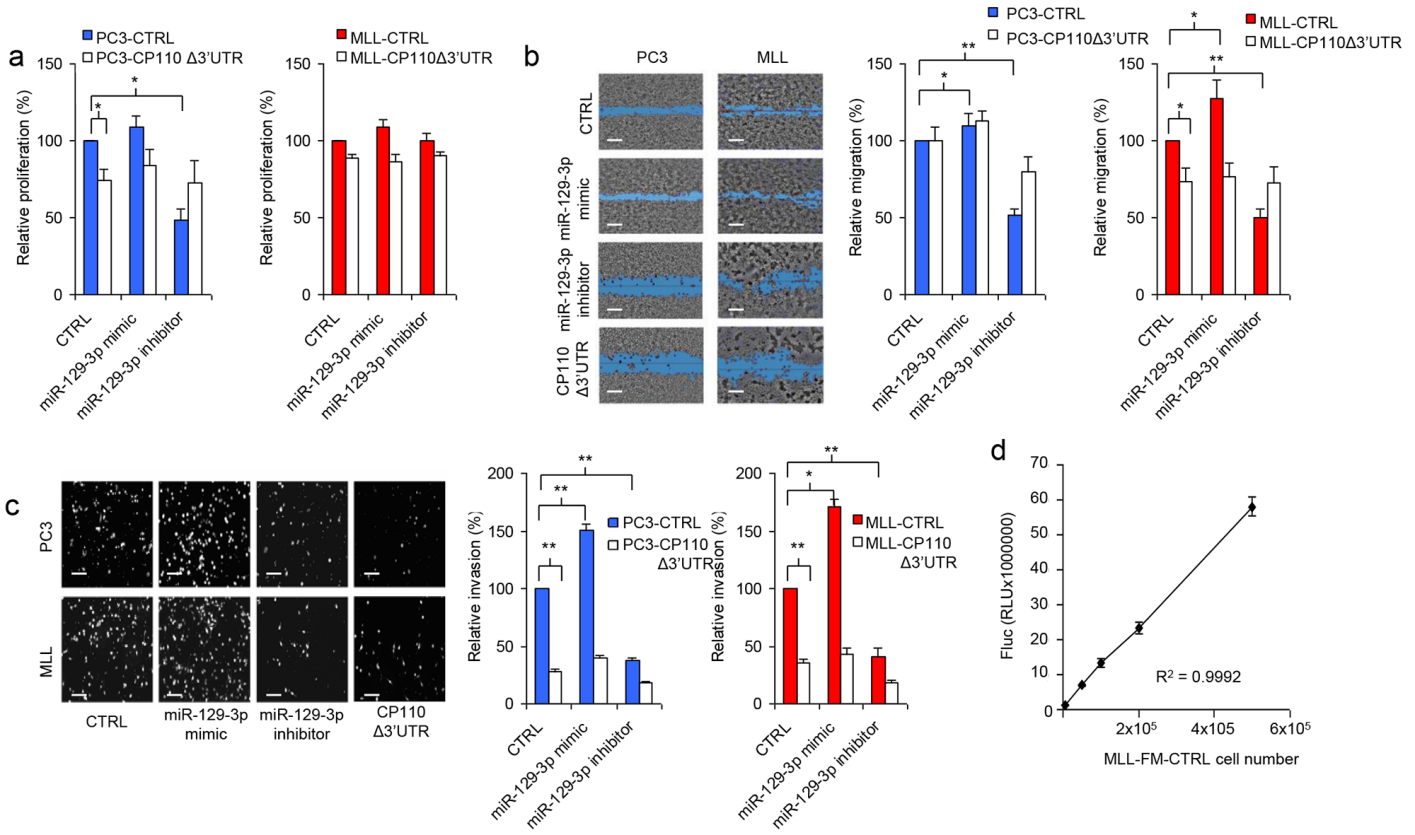
miR-129-3p and CP110 regulate cell invasion **a.** Cell proliferation analysis after overexpression of CP110Δ3′UTR and/or miR-129-3p in PC3 and MLL cells. Values represent the mean of five independent experiments ± s.e.m. **b.** Representative images of cell migration analysis after overexpression of CP110Δ3′UTR and/or miR-129-3p in PC3 and MLL cells, and quantification in the right panel. Values represent the mean of five independent experiments ± s.e.m.. Scale bar, 200 μm. **c.** Representative images of cell invasion analysis after overexpression of CP110Δ3′UTR and/or miR-129-3p in PC3 and MLL cells, and quantification in the bottom panel. Values represent the mean of five independent experiments ± s.e.m.. Scale bar, 200 μm. **d.** Fluc expression by MLL-FM-CTRL cells is linearly correlated to cell number.

**Figure 4:**
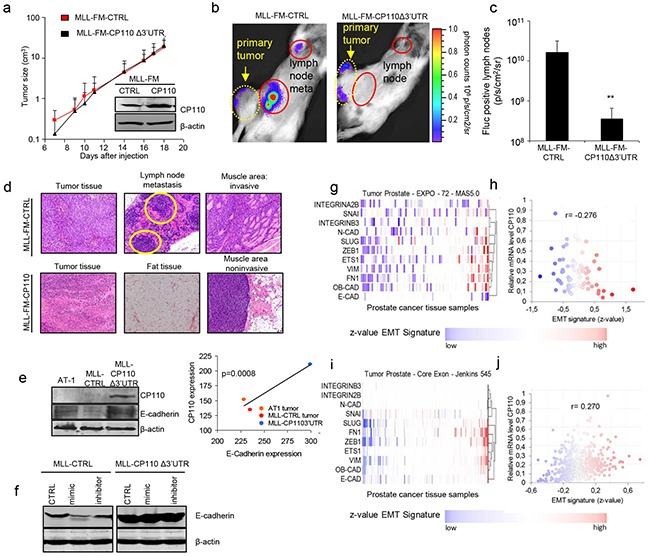
miR-129-3p and CP110 regulate cell metastasis **a.** Primary tumor growth of MLL-FM and MLL-FM-CP110Δ3′UTR cells *in vivo*. Insert shows CP110 Western blot of the injected cell cultures. **b.** Representative CCD camera images of Fluc bioluminescence imaging of lymph node metastasis, and **c.** Quantification of Fluc positive lymph nodes in MLL-FM-CTRL (n=7 rats) and MLL-FM-CP110Δ3′UTR (n=8 rats) cells. **d.** H&E staining of tumor tissues, the invaded muscle area and positive lymph node metastasis. Only for MLL-FM cells muscle invasion and lymph node metastasis (indicated by yellow circles) were detected. **e.** Western blot for E-cadherin and CP110 in tissues of different progression stages of the Dunning model. **f.** Western blot for E-cadherin in cell cultures **g-j.** Correlation of CP110 with an EMT-related signature [45] as being an averaged value of all z-values of the normalized data in two clinical PCa datasets. *p<0.05,**p<0.01, T-test.

The functional role of miR-129-3p and CP110 in PCa progression was examined by transfecting miR-129-3p mimic and inhibitor into PC3, PC3-CP110Δ3′UTR, MLL and MLL-CP110Δ3′UTR cells. The non-metastatic AT1 PCa cells do not survive in culture, therefore these cells were not included. Overexpression of CP110Δ3′UTR in PC3 and MLL cells resulted in a minor reduction in cell proliferation *in vitro* (Figure 3a), a moderate decrease in migration (Figure 3b), and a large reduction in cell invasion (Figure 3c). Overexpression of miR-129-3p in PC3 and MLL PCa cells significantly increased the invasive phenotype of these cells (p<0.01 and p<0.05, respectively), while only moderate effects on proliferation and migration were observed (Figure 3a-3c). Proliferation, migration and invasion were not affected by miR-129-3p mimics or inhibitors in cells overexpressing the CP110Δ3′UTR-rescue construct. These results suggest that miR-129-3p mediates invasion of PCa cells via repression of CP110. We injected 5×10^5^ MLL-FM-CTRL or MLL-FM-CP110Δ3′UTR cells with F-luciferase expression (Figure 3d) subcutaneously in Copenhagen rats (n=7 and n=8, respectively), and monitored tumor growth. CP110Δ3′UTR overexpression did not affect the growth rate of the primary tumor *in vivo* (Figure 4a), but did result in less invasive growth of the primary tumor as compared to the highly invasive MLL-FM-CTRL tumors (Figure 4b-4e). MLL-FM-CTRL lymph node metastases were visible by bioluminescence imaging using a CCD camera in 5 out of 7 rats (Figure 3f-3g) and confirmed by H&E staining (Figure 4d). This was in contrast to MLL-FM-CP110Δ3′UTR tumors that caused lymph node metastasis in only 1 out of 8 rats (p<0.01) (Figure 4c-4d). These observations are indicative of a key role for CP110 in PCa invasion and metastasis. PCa metastases have been associated with down-regulation of E-cadherin, Western blot analysis of E-cadherin protein expression demonstrated that MLL-FM-CP110Δ3′UTR tumor tissues with low metastatic potential had elevated levels (20%) of E-cadherin protein as compared to MLL-FM-CTRL tumor tissues with high metastatic potential (Figure 4e-4f). Moreover, the CP110 and E-cadherin expression in tissues of AT1 and MLL tumors correlated positively (r^2^=0.9117, p=0.01; Figure 4e). In addition, after transfection of MLL cells with miR-129-3p mimics we observed a decrease in E-cadherin expression in MLL cells, but not in MLL-CP110Δ3′UTR cells (Figure 4f). In order to determine whether EMT is involved in the observed PCa metastasis, we correlated CP110 gene expression to EMT-related genes in multiple independent clinical PCa datasets [23–26] (Figure 4g-4j and Supplementary Table S3). We selected a previously reported subset of EMT-related genes [27]. Surprisingly, no significant correlation was measured between CP110 mRNA expression and EMT-related genes in local and metastatic PCa tissues, suggesting that the CP110-controlled PCa cells are not undergoing EMT prior to metastasis.

### CP110 decreases PCa invasion via centrosomes

**Figure 5:**
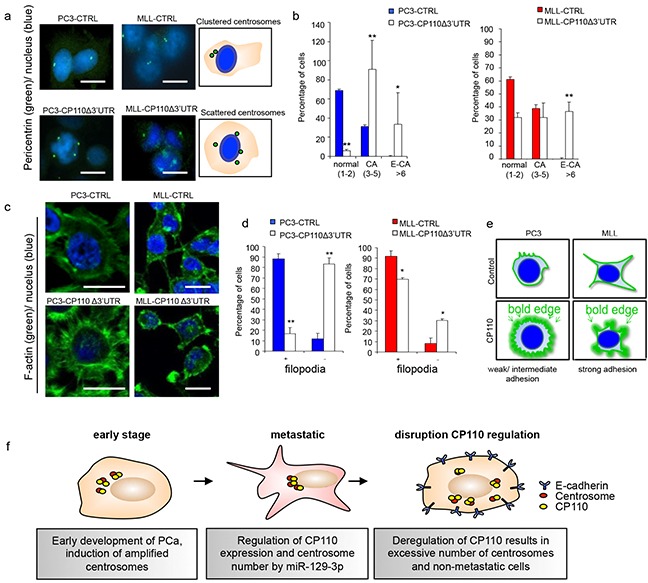
CP110 regulates PCa invasion via centrosomes **a.** Left panel cells stained for pericentrin (green) and DNA (hoechst, blue). Right panel schematic representation of pericentrin stained cells. **b.** Quantification of a). **c.** Cells stained for F-actin (green) and DNA (Hoechst, blue). **d.** Quantification of c). **e.** Schematic representation of F-actin stained cells. **f.** Schematic overview for the functions of miR-129-3p and CP110 in PCa metastasis. At an early stage, PCa cells show amplified centrosome number leading to a more aggressive phenotype. When cells enter a metastatic state, the centrosome number is regulated by miR-129-3p mediated repression of CP110. Upon overexpression of CP110, cells will increase centrosome number, E-cadherin expression leading to a non-metastatic phenotype. *p<0.05, **p<0.01; T-test.

To determine whether CP110 affected the cytoskeleton and the centrosomes in PCa cells we analysed the F-actin cytoskeleton organization and pericentrin localization in MLL, MLL-CP110Δ3′UTR, PC3 and PC3-CP110Δ3′UTR cells. CP110 plays a key role in centrosome organization and its overexpression may cause aberrant centrosome function due to E-CA, which may decrease cell invasion and metastasis. E-CA was evaluated by staining MLL, MLL-CP110Δ3′UTR, PC3 and PC3-CP110Δ3′UTR cells for the centrosomal marker pericentrin (Figure 5a-5b). A regular cancer associated centrosome phenotype was found, with modest CA for PC3 and MLL. By contrast, the centrosomes moved towards an E-CA phenotype in MLL-CP110Δ3′UTR and PC3-CP110Δ3′UTR cells (Figure 5a-5b). The E-CA of centrosomes in MLL-CP110Δ3′UTR and PC3-CP110Δ3′UTR cells indicates an uncontrolled centriolar duplication rather than centrosome fragmentation due to structural instability [28]. Furthermore, centrosomes were clustered in control cells, while centrosomes were scattered in CP110Δ3′UTR overexpressing cells, which is also indicative for disrupted directional movement of cells [29]. To study whether the scattered overduplicated centrosomes affected cell directional movement, we stained MLL, MLL-CP110Δ3′UTR, PC3, and PC3-CP110Δ3′UTR cells for F-actin and determined cytoskeleton structure. We observed a clear decrease in F-actin expression. Furthermore, in MLL-CP110Δ3′UTR cells, the long F-actin branching structures were deregulated, with a rounded edge surface, compared to the control cells (Figure 5c-5e). This is in agreement with the observed increase in E-cadherin expression (Figure 4f) and decrease in cell invasion (Figure 3c). The distinct cytoskeleton phenotypes (Figure 5c-5e) are consistent with rescue of CP110 expression changing the F-actin distribution to a 'bold-edge' phenotype. In conclusion, our results indicate that miR-129-3p is upregulated in metastatic PCa cells resulting in repression of CP110, which is accompanied by loss of E-cadherin expression, cytoskeleton remodelling, and the formation of filopodia, endowing PCa cells with increased migration and invasion capacity (Figure 5f).

## DISCUSSION

In early stages of cancer development, CA is a key contributor for development into more aggressive types of cancer [30]. However, in the present study we show that when aggressive prostate cancer cells acquire metastatic capacity, a feedback loop is activated that controls centrosome number. We identified miR-129-3p to be upregulated in metastatic prostate cancer cells, which negatively regulates CP110 expression. Rescue of CP110 expression in metastatic PCa cells caused E-CA and prevented invasion, and lymph node metastases. About 50% of the miRNAs that were upregulated in metastatic PCa MLL have a binding site in the 3′-UTR of CP110, which suggests that simultaneously with miR-129-3p other miRNAs may also contribute to the control of CP110 levels and centrosome function. miR-34 was previously confirmed to repress CP110 [19] and is also strongly upregulated in metastatic PCa. We do not exclude a role for miR-34 or other miRNAs in the repression of CP110 in metastatic PCa (Supplementary Table S2), however functional experiments with miR-129-3p inhibitors clearly demonstrate a significant contribution of miR-129-3p to the expression of CP110.

We confirmed that PCa metastasis is accompanied by down-regulation of E-cadherin [31], resulting in cytoskeleton rearrangements and the formation of filopodia, typical for EMT [31]. Down-regulation of E-cadherin is an important hallmark of EMT, a process involved in the first steps of the tumor metastasis cascade [31, 32]. Although we observed a direct correlation between PCa metastasis, CP110 expression, and E-cadherin expression, we found no significant correlation between CP110 expression and the expression of EMT-related genes in four independent clinical PCa datasets. Hence, we suggest that centrosomal CP110 controls the metastatic process in PCa cells in an EMT-independent manner, as was recently also postulated for the role of centrosomes in the formation of breast cancer metastases [5]. CA is described to occur at early stages of cancer progression and has been related to increased tumor aggressiveness [4, 33, 34]. However, at a later stage in the metastatic process the relation between amplified centrosomes and metastasis remains unclear. Reports in which a relation between CA and metastasis is studied are conflicting [35]. CP110 expression has previously been described to lead to amplified centrosomes, and increased cell invasion *in vitro* [10]. However, the nature of CP110 and CA regulation in metastatic cancer has not been described. We demonstrate that CA (3-5 centrosomes/cell) in PC3 and MLL cells is regulated through control of CP110 expression by miR-129-3p. CP110 overexpression and abrogation of its regulation by deletion of miR-129-3p binding site in CP110-3′UTR, resulted in an excess amplification of centrosomes, and reduced aggressiveness of prostate tumor cells. Most important for cancer cells to maintain their polarized potential is to correctly position the centrosomes within the cell [36]. This positioning is regulated by multiple proteins [36]. Moreover, an excess number of centrosomes can lead to disruption of cell migration by centrosome scattering [37, 38]. In agreement with this, we observed a lack of centrosome clustering in CP110 overexpressing PCa cells in vitro and reduced metastatic potential *in vivo*. Importantly, the centrosome is emerging as a potential therapeutic target in cancer, including PCa [39]. Potential centrosomal drug targets include cyclin-dependent kinases, polo-like kinases [40–42], aurora kinases [43] and molecular motor proteins [44]. Several small molecule inhibitors affecting centrosome function have been reported, of which Plk1 inhibitors are already tested in initial clinical studies [40, 45, 46]. While CP110 may also regulate ciliation, a process usually downregulated in (prostate) cancer cells [47], it remains to be investigated to what extent dysfunctional cilia contribute to PCa metastasis and the undirected migration of PCa cells.

In conclusion, we demonstrate that the centrosomal protein CP110 is at least partly regulated by miR-129-3p, and plays a functional role in PCa invasion and metastasis. Our results suggest that in metastatic PCa cells miR-129-3p is upregulated to reduce CP110 levels, preventing cells to develop an excess number of centrosomes. These results provide a functional link between aberrant centrosome function and PCa metastasis. Further research aimed at therapeutic targeting of the centrosome in metastatic PCa cells is warranted.

## MATERIALS AND METHODS

### Cells

Three stages of the Dunning model were selected for miRNA profiling studies, i.e. anaplastic tumor R3327-AT (AT1; invasive growth, non-metastatic) and R3327-MATLyLu tumors (MLL; metastatic) (Figure 1A). The tumor cells were expanded in male Copenhagen rats *in vivo* [48]. For *in vitro* studies, MLL and the human prostate cancer cell lines PC3 and LNCaP (ATCC) were cultured in RPMI 1640 culture medium, supplemented with 10% FCS and 50 U/ml penicillin and 50 μg/ml streptomycin. All cells were maintained in a 5% CO_2_-humidified atmosphere at 37°C. MLL-FM-CTRL cells were engineered by transduction with a lentivector encoding Firefly luciferase and mCherry as described elsewhere [49]. MLL-FM-CP110Δ3′UTR cells were generated by stable transfection and selection of MLL-FM-CTRL cells with a CP110 rescue expression construct without miR-129-3p 3′-UTR binding site (OriGene, Rockville, MD). PC3-CP110Δ3′UTR cells were generated with the same CP110 rescue construct.

### miRNA profiling

miRNA profiling of AT1 and MLL tumor tissue (n=4) was performed by Exiqon (Vedbaek, Denmark). RNA was isolated from the tumor homogenates by Qiagen miRNA isolation kit (Qiagen). Total RNA quality was verified by an Agilent 2100 Bioanalyzer profile. 1 μg total RNA from sample and reference was labelled with Hy3 and Hy5 fluorescent label, respectively, using the miRCURY LNA Array power labelling kit (Exiqon, Denmark). The Hy3-labeled samples and a Hy5-labeled reference RNA sample were mixed pair-wise and hybridized to the miRCURY LNA *Array version 5^th^*Generation** (Exiqon), which contains capture probes targeting 387 miRNAs for rat registered in miRBASE 15.0. The hybridization was performed using a Tecan HS4800 hybridization station (Tecan, Austria). The slides were scanned with the Agilent G2565BA Microarray Scanner System (Agilent Technologies, Inc., USA) and image analysis was carried out using the ImaGene 9.0 software (BioDiscovery, Inc., USA). The quantified signals were background corrected (Normexp with offset value 10) and normalized using the global Lowess (Locally Weighted Scatterplot Smoothing) regression algorithm. miRNAs were selected for significant (p<0.05) up- or downregulation. RNA sequencing of human miRNAs expressed in cell cultures was performed according to Illumina protocols, as previously described [50]. For data analysis, we applied Illumina's software packages (SCS2.9/RTA1.9 and Off-line Basecaller v1.9).

### Patient archival prostate tissue samples

Prostate tissues from 41 patients with adenocarcinoma of the prostate were evaluated for miRNA expression levels. The diagnosis was based on histopathological examination. Radical prostatectomies were performed between 1994 and 2011 at the Department of Urology, VU University Medical Center, Amsterdam, The Netherlands. Selection criteria were tumor present in the tissue blocks and prognosis. Postoperatively, the patients were scheduled for regular follow-up visit at the institutional outpatient clinic. Biochemical progression was defined as rising postoperative PSA levels by >0.2 ng/ml, confirmed in a consecutive visit. Biochemical recurrence was observed in 25 of the total of 41 patients.

### Transfection with miRNA mimics and inhibitors

Cells were transfected by reverse transfection of miRNA precursors (mimics) or inhibitors or their controls (20 nM in optiMeM, Ambion, Austin), according to the manufacturer's instructions. For each experiment, the media were removed 24 h after transfection and replaced by regular culture media. The cells were cultured for another 24 h prior to use in experiments.

### Proliferation assay

Cells were seeded (2,000 cells/well) for reverse transfection in 96-well plates (Greiner BioOne, Frickenhausen Germany). At 24 h after transfection, the medium was replaced by fresh culture medium. After 72 h the cells were fixed by trichloroacetic acid at 4°C for 1 h and stained by sulphorhodamine B (SRB). The SRB-dye was diluted in 10 mM TRIS solution and the optical density (OD) was measured at 492-540 nm after which the OD of the controls was set to 100%.

### Migration assay

At 24 h after reverse transfection, the medium was replaced by fresh culture medium and 24 h later, the wound healing (scratch) assay was performed after uniformly scratching cellular monolayers using a guided 96-well pin tool (Peira, Turnhout, Belgium). Wells were washed, and fresh medium was added. Images were automatically captured at t=0 and t=6 h on a Leica DMI3000 microscope (Leica, Rijswijk, The Netherlands) using Universal Grab 6.3 software (DCILabs, Keerbergen, Belgium). Scratch sizes were determined using Scratch Assay 6.2 (DCILabs). Wound closure (μm^2^) was expressed as a percentage of that in control wells.

### Invasion assay

The invasion assay was carried out using transwell chambers with a fluorescence-blocking 8 μm pore filter insert (Becton Dickinson Labware, Bedford, MA, USA) as described previously [51]. In brief, the insert was coated with 5 ng matrigel (Sigma-Aldrich Chemicals, Zwijndrecht, The Netherlands). Cells (200,000/insert) were seeded 48 h after transfection in serum free medium. In order to count the cells that have invaded after 24 h, calcein-AM fluorescently labelled cells were counted.

### RT-PCR

RNA from cell lines and frozen tumor tissues was extracted using a microRNeasy kit (Qiagen, Westburg, Leusden, The Netherlands) according to manufacturer's instructions. RNA for paraffin embedded tissues was extracted using Qiagen FFPE RNA isolation kit according to manufacturer's instructions. Subsequently, extracts were reverse transcribed using MultiScribe Reverse Transcriptase and the specific miRNA primer (Life Technologies) according to manufacturer's instructions. cDNA samples were amplified using a LightCycler 480 (Roche Diagnostics, Almere, The Netherlands) with 15 sec 95°C denaturation and 60 sec 60°C primer annealing/extension for 50 cycles starting with a 10 min hot start at 95°C. In relative PCR quantifications, RNU-6B was used as endogenous control, and results are expressed in arbitrary units fold change compared to AT-1-tumor or LNCaP cells.

### Western blotting

Whole cell protein was isolated by scraping in lysis buffer (Cell Signaling Technology, Inc, Danvers, MA, USA). Western blot was performed as described previously [51]. Briefly, from each condition 30 μg of protein was separated on a 10% SDS-PAGE and electroblotted onto polyvinylidenedifluoride (PVDF) membranes (Millipore Immobilon-FL PVDF, 0.45 μm). Subsequently, the membranes were blocked and incubated overnight at 4°C with the anti-CP110 (Cell Signaling, 1:1,000), E-cadherin (1:500; Novacastra, UK) or anti-β-actin-antibody (Sigma, dilution 1:10,000). The bands were scanned using an Odyssey Infrared Imager (Westburg), 84 μm resolution, 0 mm offset and with high quality.

### Immunofluorescent staining

In 8-chamber labtek microscope slides, 100,000 cells were seeded. After 48 h cells were fixed in ice-cold methanol and blocked 30 min with 3% BSA (room temperature (RT)) and stained for either F-actin (Sigma) or pericentrin (provided by C. King, VU University, Amsterdam, NL) overnight at 4°C. After washing, the secondary antibody was incubated (1:200, anti-mouse-FITC, Dako) for 1 h at RT. The nucleus was stained with Hoechst 33342 (1:2,000) for 10 min at RT. Subsequently, slides were washed, and mounted onto microscope slides using vectashield (Vector, Burlingame, CA, USA). Stained cells were imaged on a Zeiss confocal LSM510 microscope (Carl Zeiss, Jena, Germany) using a x40 oil objective (NA=1.20). Imaged were analysed by Fiji software (Fiji, www.fiji.sc).

### *In vivo* metastasis

Animal experiments were approved by the local committee on Animal Experiments of the VU University Medical Center, Amsterdam, the Netherlands. Male Copenhagen rats (age 2 months) were injected subcutaneously at the flank of the lower back with 500,000 cells/200 μl of either MLL-FM-CTRL (n=7 rats) or MLL-FM-CP110Δ3′UTR (n=8 rats) cells. Primary tumor growth was measured using a calliper and the volume was calculated using the formula: (length x width^2^)/2. In addition presence of lymph node metastasis was measured weekly by measuring the Fluc signal by a charge-coupled device (CCD) camera, using the Xenogen-IVIS Lumina System (Xenogen Corp., Alameda, CA). Rats were injected intraperitoneally with 300 μl D–Luciferin (200 mg/kg body weight) under anaesthesia (1.5 L O_2_/minute and 3% isoflurane). Regions of interest were defined in the abdomen where lymph node metastases were expected of which the local bioluminescence signals and background signals were subtracted. The photon counts (p/s/cm^2^) in these regions were used as a total measurement of Fluc activity.

### Immunohistochemical staining of CP110

Immunohistochemistry was performed on 3 μm thick paraffin embedded sections of radical prostatectomy specimens. Slides were deparaffinised by steps of xylene and subsequently rehydrated by decreasing concentrations of ethanol. Endogenous peroxide expression was blocked by incubation with 0.3% H_2_O_2_ in methanol for 30 min. Slides were heated for antigen retrieval in 1 mM Citrate buffer (pH 6.0). Slides were blocked with 3% BSA for 10 min at RT and exposed to the primary antibody against anti-CP110 (1:200, O/N at 4°C). After incubation, sections were washed and incubated with post-antibody blocking solution (Immunologic, Duiven, The Netherlands) for 15 min at RT. Subsequently, slides were washed and incubated with Brightvision-plus (Immunologic) for 60 min at RT. Peroxide was revealed by AEC solution (Invitrogen). Slides were counterstained with Hematoxylin and were covered in Kaisers glycerol (Merck KGAa, Darmstadt, Germany). Blinded for the clinical parameters, including Gleason, progression and metastasis development, immunohistochemically stained slides were reviewed by both an experienced pathologist and senior researcher. CP110 staining of the tumor tissue section was scored for staining intensity (0-3).

### EMT analysis

For analysis of the relation between CP110 and EMT genes, a previously reported EMT marker gene set was selected [27]. From this a weighed and directional gene signature was created using the appropriate prostate tumor, MAS5.0 normalized, mRNA expression sets using the R2 program (http://r2.amc.nl). Expression of CP110 expression was correlated with this weighed and averaged signature score. Datasets that were selected for analysis include: ExpO (http://www.intgen.org), Jenkins-545 [23], Sueltman [24], Ethnic-89-MAS5.0-u133a [25] and Svensson-108-MAS5.0-u133a [26].

### Statistical analysis

For calculating significant differences between the parental and the transfected cells or between treated and untreated samples, the two-tailed Student's t-test was used. The values were considered significantly different when p<0.05. *Chi*-square test was used for calculation of significant differences between patient groups of metastasis and non-metastasis and/or high and low Gleason score, using SPSS 20.0. Groups were found to be statistically different when p<0.05.

## SUPPLEMENTARY TABLES








