# Supplementary material for: miR-129-3p controls centrosome number in metastatic prostate cancer cells by repressing CP110
Source: Oncotarget. 2016 Feb 23;7(13):16676–87. doi: 10.18632/oncotarget.7572 (PMC4941343; doi:10.18632/oncotarget.7572)
Supplement: Supplementary file 1 [file oncotarget-07-16676-s001.pdf]

SUPPLEMENTARY TABLES

Supplementary Table S1: miRNA expression comparing MLL to AT-1.

See Supplementary File 1

Supplementary Table S2: Analysis of miRNAs targeting CP110 comparing 10 databases.

See Supplementary File 2

Supplementary Table S3: Correlation between CP110 expression and EMT signature.

See Supplementary File 3
